# Response of yield and quality of giant embryo rice to nitrogen application and analysis of lipid-lowering effect

**DOI:** 10.3389/fpls.2022.1023677

**Published:** 2022-10-06

**Authors:** Mingming Hu, Guangyi Chen, Ligong Peng, Congmei Li, Xingmei He, Qiuqiu Zhang, Hong Yang, Chaode Liang, Hudong Kuang, Yan Lan, Tian Li

**Affiliations:** ^1^ Crop Ecophysiology and Cultivation Key Laboratory of Sichuan Province, College of Agronomy, Sichuan Agricultural University, Chengdu, China; ^2^ College of Life Science and Engineering, Southwest University of Science and Technology, Mianyang, China

**Keywords:** giant embryo rice, nitrogen application rate, yield and quality, hyperlipidemia, gamma-aminobutyric acid

## Abstract

Giant embryo rice is known as a highly nutritious functional rice because it is rich in gamma-aminobutyric acid (GABA), which has various regulatory functions in the human body. To study the response of giant embryo rice yield and quality to nitrogen (N) application, and to verify the effect of giant embryo brown rice on alleviating hyperlipidemia in rats. In this study, field experiments were conducted in 2020 and 2021 using the giant embryo rice varietiers J20 (japonica) and Koshihikari (japonica) rice as experimental materials and five N levels, 0 (N_0_), 90 (N_1_), 135 (N_2_), 180 (N_3_) and 225 (N_4_) kg ha^-1^. The results showed that the yield of both varieties increased with increasing N and the maximum values were observed under the N_2_ treatment. As more N was gradually applied, the brown rice rate, milled rice rate, head rice rate and GABA content of both varieties first increased and then decreased, while the chalky grain rate and chalkiness showed the opposite trend. The optimal values of these indexes were observed under the N_2_ treatment. The peak viscosity and breakdown value of J20 decreased, while its setback value and pasting temperature increased with increasing N. In contrast, Koshihikari showed the opposite trend. The protein content and protein component contents of both varieties showed an increasing trend with increasing N, among which gliadin was the most sensitive protein component to N fertilizer. Animal experiments results showed that J20 brown rice could significantly slow the rate of weight gain of rats, reduce serum total cholesterol and triglyceride levels and increase high-density lipoprotein cholesterol levels. Therefore, increasing N could effectively enhance J20 yield and improve processing, appearance and nutritional quality but decrease cooking and eating quality. The brown rice J20 had the effect of slowing the rate of weight gain and reducing the hyperlipidemia level of rats, the optimal N application rate for achieving high yield, high quality and good functional characteristics in the giant embryo rice J20 was 135 kg ha^-1^. These findings will provide a theoretical and technical foundation for the popularization and application of giant embryo rice in the future.

## 1 Introduction

Giant embryo rice is a special, highly nutritious, multifunctional rice; its embryo is 2-3 times larger than that of ordinary rice, and some embryos can even reach more than 5 times the size of those of ordinary rice (Wei et al., 2005; Fang et al., 2021). Rice embryos are rich in natural nutrients such as protein, lipids, and vitamins, especially GABA, which has various regulatory functions in the human body (Zheng et al., 2012; Peng et al., 2019). The functions of GABA include lowering blood lipids, activating the liver and kidney, and preventing obesity; moreover, giant embryo rice is known as “longevity rice” and “high-nutrition functional rice” (Chung et al., 2021). Hypertension is one of the most common lifestyle-related diseases. Some studies have shown that the use of giant embryo rice can alleviate the high cholesterol, high blood sugar and other diseases (Hee-Ky et al., 2020). Feeding the mice with giant embryo rice, it can effectively prevent diabetes and hyperlipidemia, and has the effect of reducing body weight (Chung et al., 2021). Therefore, the giant embryo rice has attracted increasing attention from rice genetic and breeding experts at home and abroad in recent years because it meets people’s current food consumption concept and healthy life concept.

However, there is limited research on the supporting cultivation technology of giant embryo rice, especially the effects of different N application rates on GABA content. It mainly involved areas such as the innovation of germplasm resources (Zhang et al., 2017) and nutritional value analysis (Zhang et al., 2013). Many giant embryo rice varieties, such as “Haiibuki” (Kawakami et al., 2018), “giant embryo rice TgeB” (Zhang et al., 2008), and “Shangshida No. 5” (Ren et al., 2011), have been cultivated at home and abroad. The contents of protein, lipids, minerals, vitamins and amino acids in giant embryo brown rice have been increased to varying degrees (Zhang, 2008), especially the GABA content, which has been increased significantly, and in some varieties the GABA content is even 2-6 times higher than that in ordinary rice (Yang, 2008). Nitrogen is one of the most important limiting factors in rice production and plays an important role in rice growth and development and yield and quality improvement (Pan et al., 2017). For a long time, the application of nitrogen N fertilizer has been an important measure to ensure high rice yield and quality. However, excessive application of N will not only reduce rice yield and quality but also cause environmental pollution (Ju et al., 2015). Therefore, the appropriate N application rate is the key factor to ensure the high-quality cultivation of giant embryo rice.

Giant embryo rice is still planted sporadically in China. On the one hand, this is due to the lack of varieties with high yield, high quality and wide adaptability; on the other hand, there is no mature cultivation technology, and making it difficult to widely promote giant embryo rice cultivation (Dai et al., 2011). Therefore, an experiment was conducted involving the giant embryo rice J20 and five N application levels. The objective of this study was to determine the effects of N application on the yield and quality of giant embryo rice and to explore the effects of giant embryo rice on hyperlipidemia model rats. The results of this study will provide a theoretical and technical foundation for the popularization and application of giant embryo rice.

## 2 Materials and methods

### 2.1 Experimental site information

The experiment was conducted in Chongzhou Modern Agricultural Research Park of Sichuan Agricultural University from 2020 to 2021 (30°56′ N, 103°65′ E). The previous crop was rapeseed, and the texture of the topsoil (0-20 cm) was sandy loam. Organic matter (K_2_Cr_2_O_7_ -volumetric method), total nitrogen (Kjeldahl method, UDK-169, ITA), alkaline hydrolyzable nitrogen (alkali hydrolysis diffusion method), available phosphorus (Mo–Sb colorimetry after digestion with H_2_SO_4_ and HClO_4_), available potassium (flame spectrometry after NH_4_OAc extraction), pH (tested in a sample containing a 1:2.5 ratio of soil to water). The basic fertility of the tested soil is shown in **Table 1**.

**Table 1:** The basic fertility of the tested soil.

Year	Organic matter(g kg^-1^)	Total nitrogen(g kg^-1^)	Alkaline hydrolyzable nitrogen(g kg^-1^)	Available phosphorus(mg kg^-1^)	Available potassium(mg kg^-1^)	pH
2020	21.50	1.91	110.54	18.91	58.31	5.93
2021	24.50	1.72	108.35	19.75	60.24	6.04

### 2.2 Experimental materials

**Figure 1:**
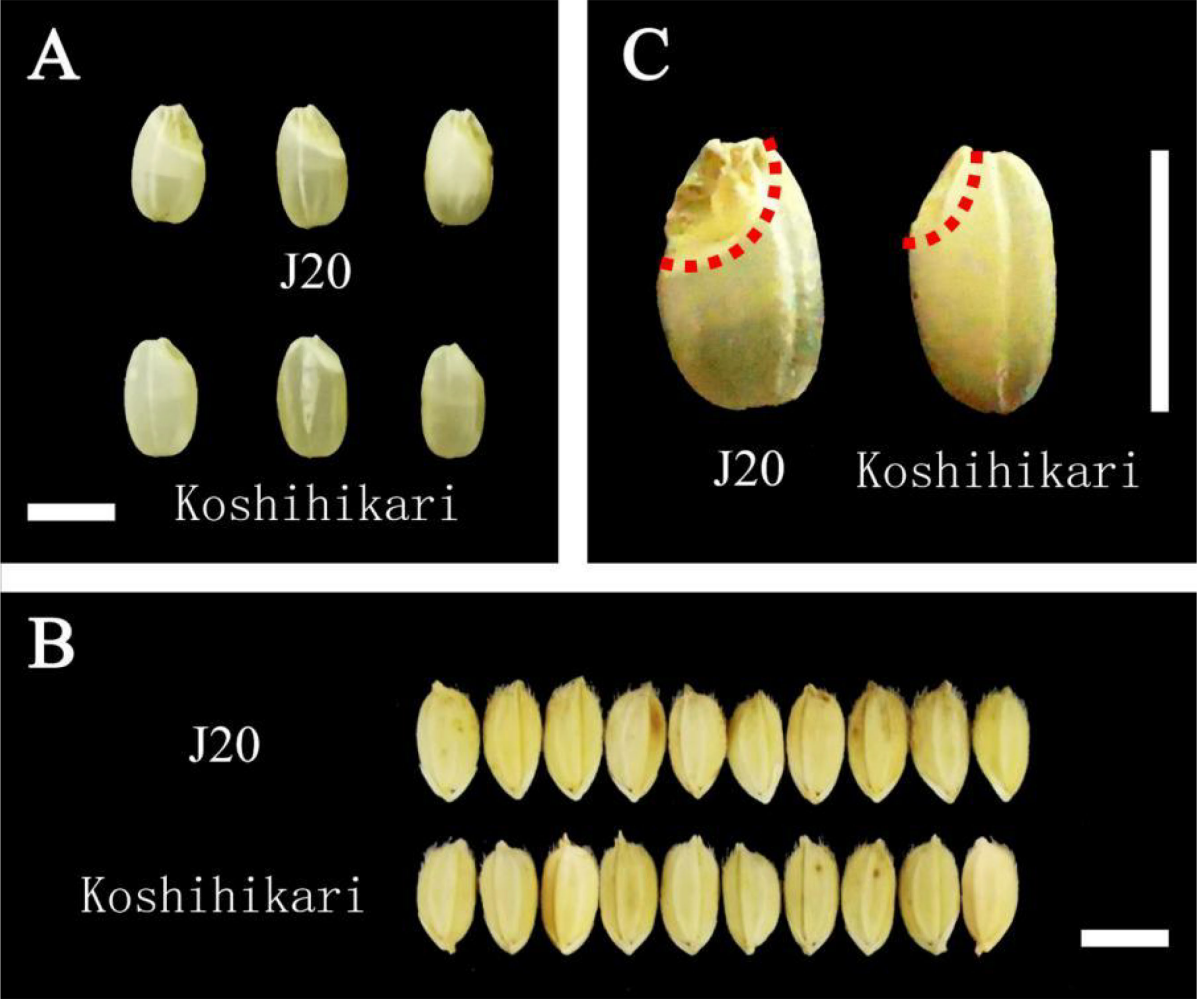
Comparison of grain types between J20 and Koshihikari. **(A)** Comparison of J20 and Koshihikari brown rice; **(B)** Comparison of J20 and Koshihikari grains; **(C)** J20 compared with Koshihikari brown rice embryo. The scale bar is 5 mm.

The tested rice varieties were giant embryo japonica rice J20 (selected from Koshihikari by using the chemical mutation method, and the embryo was twice as large as that of Koshihikari) and Koshihikari (Kyushu University and Agricultural Biological Resources Research Institute of Japan), both with high yield and good quality. A comparison between J20 and Koshihikari brown rice is shown in **Figure 1**.

### 2.3 Experimental design

The experiment adopted a randomized block design with 5 N levels: N_0_: 0 kg ha^-1^, N_1_: 90 kg ha^-1^, N_2_: 135 kg ha^-1^, N_3_: 180 kg ha^-1^, N_4_: 225 kg ha^-1^; N_0_ was used as the control. The plot area was 5.0 m × 4.0 m, which was replicated 3 times for a total of 30 plots. The transplant density was 30 cm × 25 cm, and 2 seedlings were planted in each hill. In 2020, the seedlings were sown on April 12, artificially transplanted on May 16, and harvested on August 25; in 2021, they were sown on April 16, artificially transplanted on May 20, and harvested on August 29. The N fertilizer used was urea (N ≥ 46%), which was applied according to a base fertilizer:tiller fertilizer ratio of 6:4. The potassium fertilizer used was potassium chloride (K_2_O ≥ 60%, 180 kg ha^-1^), and the phosphate fertilizer used was superphosphate (P_2_O_5_ ≥ 12%, 90 kg ha^-1^). The base fertilizer and all phosphorus and potassium fertilizers were applied 1 day before transplanting, and all other field management measures remained the same.

### 2.4 Sampling and measurements

#### 2.4.1 Yield and yield components

At the maturity stage, 30 hills were selected from each plot to investigate the number of effective panicles, and 5 hills were selected from each plot according to the average number of effective panicles. The total number of grains per panicle, number of grains per panicle, 1000-grain weight, seed setting rate, theoretical yield and other indicators were determined. Rice was then harvested in different areas and dried in the sun, and the actual yield was measured when the water content of rice was 14%.

#### 2.4.2 Quality

The quality of the rice of each treatment was measured after it was dried and stored for 3 months. The brown rice rate, milled rice rate, head rice rate, chalky grain rate and chalkiness degree of rice were determined in accordance with the national standard of the People’s Republic of China (GB/T 17891-2017).

A total of 3.00 g brown rice flour and 25.0 mL of distilled water were added to a test tube. Pasting properties were measured by using a rapid visco analyzer (RVA) device (3-D, Newport Scientific, Sydney, Australia) and analyzed with Thermal Cycle for Windows (TWC) software. Viscosity values were measured in a rapid viscosity analyzer unit (RVU).

The protein content was measured from the total N content of head rice with a conversion index of 5.95 *via* the Kjeldahl method.

The determination of the protein content was performed by accurately weighing 0.30 g of brown rice flour and continuously extracting different components using four solvents: ultrapure water, 0.6 mol L^-1^ NaCl, 80% ethanol and 0.06 mol L^-1^ NaOH. The absorbance value was detected at 595 nm after color development with the Coomassie brilliant blue method, and the content of each protein component of grain samples was calculated according to the standard curve (Lan et al., 2021).

#### 2.4.3 GABA content

For each treatment, an appropriate amount of brown rice grains was crushed and sieved, and then 0.4 g was accurately weighed into a 15-ml centrifuge tube. Then, 8 ml of 0.1 mol L^-1^ hydrochloric acid was added, and the mixture was shaken by hand and left to leach overnight. The mixture was then shaken on a shaker for 60 min. Next, 2 ml of supernatant was collected and centrifuged (14,500 rpm at 4°C for 15 min). Then, 500 μl of supernatant was collected, an equal volume of 10% sulfosalicylic acid was added, mixed well, and the mixture was allowed to stand at 4°C for 15 min to precipitate. Next, 500 μl of the supernatant was collected with a 1-ml syringe, filtered through a 0.45-μm filter, and detected by an amino acid analyzer (A300, Membrapure, Hennigsdorf, Germany).

#### 2.4.4 Animals and diet

All rats were purchased from Chongqing Tengxin Biotechnology Co., Ltd. with production license scxx (Yu) 2012-0008. Both J20 brown rice and Koshihikari brown rice were treated with an N application rate of 135 kg ha^-1^. Thirty 7-week-old male hyperlipidemia model rats were selected and fed with standard mouse daily chow and free access to food and water before the start of the experiment. After 7 days of adaptation, they were randomly divided into a control group (normal feed), experimental group 1 (Koshihikari brown rice) and experimental group 2 (J20 brown rice), with 10 animals in each group. All experimental animals were kept in the animal room of the College of Animal Science and Technology, Southwest University of Science and Technology. The animal room was clean and well ventilated, the temperature was 22°C ± 2°C, the relative humidity was 60% ± 5%, and the light cycle was 12 h. The feeding cycle was 5 weeks, and the weight was weighed every weekend. After the test period, the rats were anesthetized with ether following a 12-h fast. Blood samples were collected from the orbit of the rat and placed in a 10 ml vacuum blood collection tube, and centrifuged at 5000 r/min at 4°C for 10 min to separate the plasma. Then, the supernatant was dispensed into 2 ml centrifuge tubes and stored at -60°C for later use. An automatic biochemical analyzer was used for the determination of triglyceride (TG), total cholesterol (TC), high-density lipoprotein cholesterol (HDLC) and low-density lipoprotein cholesterol (LDLC).

### 2.5 Statistical analysis

Data were analyzed by using analysis of variance (ANOVA), and means were compared based on the least significant difference (LSD) test at the 0.05 probability level by using SPSS 25.0 (Statistical Product and Service Solutions Inc., Chicago, IL, USA). Origin Pro 2020 (OriginLab, Northampton, MA, USA) was used to draw the figures.

## 3 Results

### 3.1 Yield and yield components

**Figure 2:**
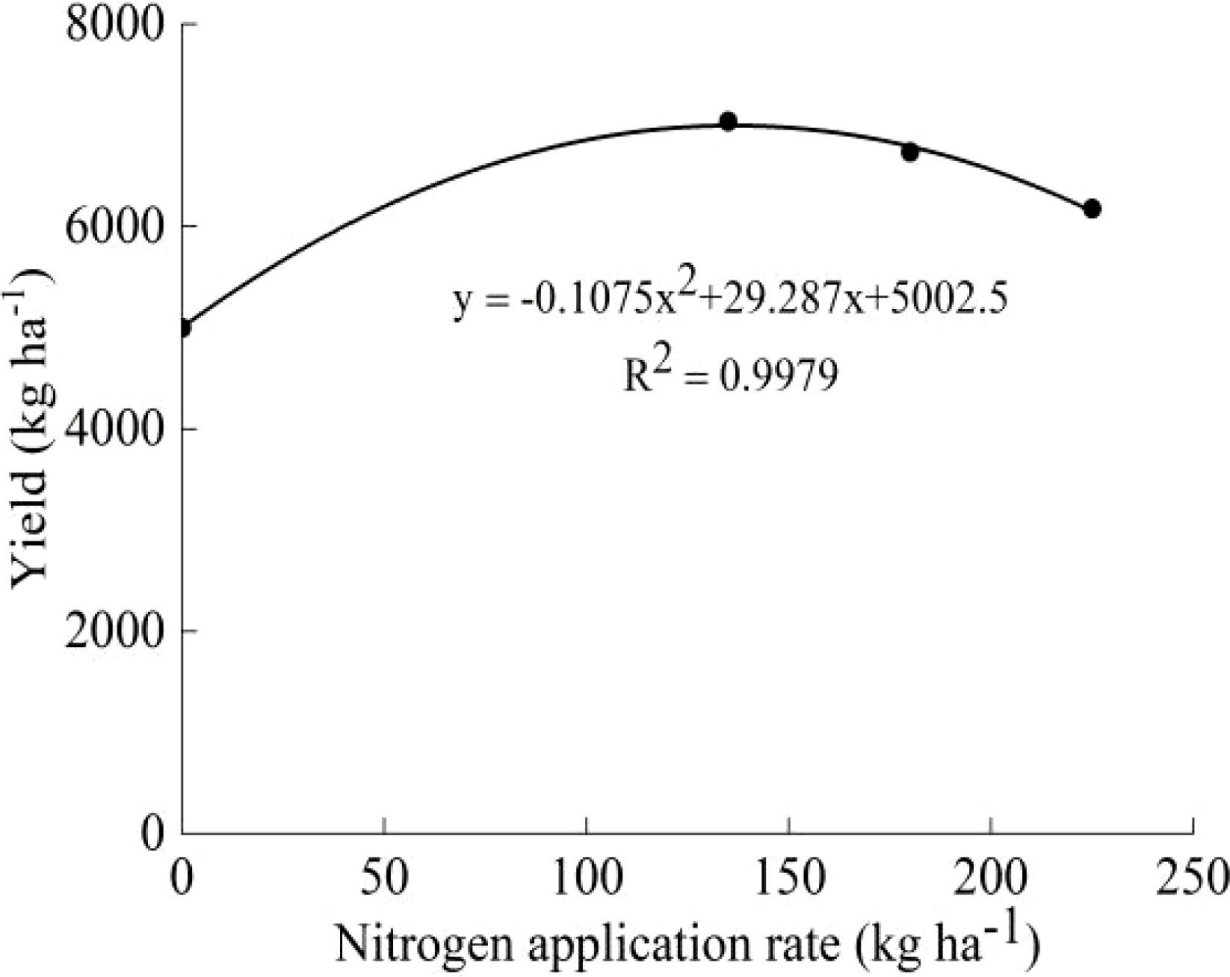
Relationship between J20 yield and nitrogen application rate.

**Figure 3:**
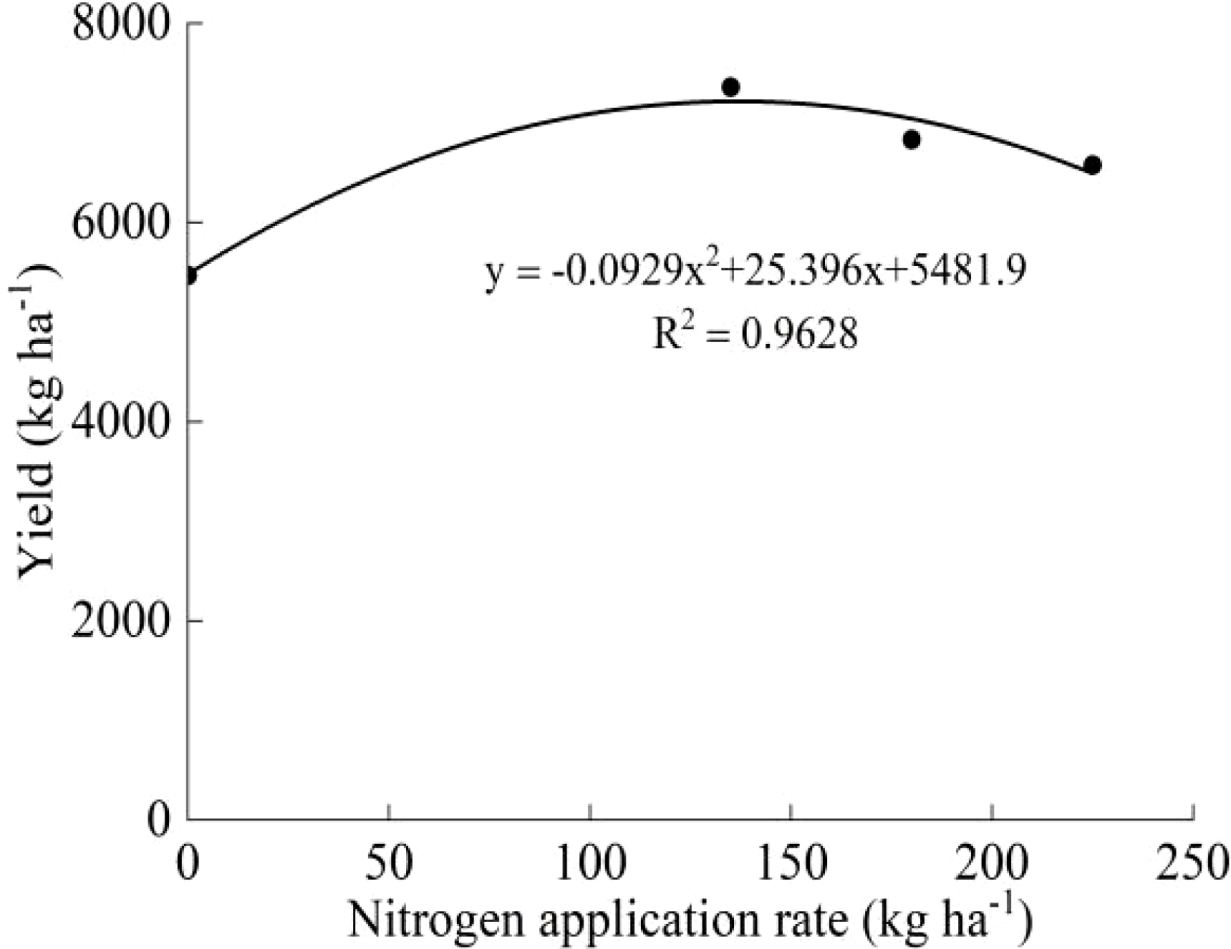
Relationship between Koshihikari yield and nitrogen application rate.

The two-year yields of the two varieties showed a trend of first increasing and then decreasing with increasing N, and the highest yield was observed under the N_2_ treatment (**Table 2**). The average yield of the two varieties under the N_2_ treatment increased by 40.74% (J20) and 34.55% (Koshihikari) compared with that under the N_0_ treatment. There was no significant difference among the yields under the N_2_, N_3_, and N_4_ treatments, but they were all significantly higher than that under the N_0_ treatment. In the two-year experiment, the J20 yield had the largest variation coefficient and was more sensitive to N fertilizer, but the yield of J20 was lower than that of Koshihikari under the same N application rate. According to the average yield and N application rate of J20 and Koshihikari in the two years, the effect equations were established. For J20, the optimum N application rate was 136.22 kg ha^-1^ and the theoretical maximum yield was 6997.22 kg ha^-1^ (**Figure 2**). For Koshihikari, the optimum N application rate was 136.68 kg ha^-1^, and the theoretical maximum yield was 7217.52 kg ha^-1^ (**Figure 3**).

**Table 2:** Effects of N application rate on rice yield and yield components.

Year	Variety	Treatment	Effective Panicles(×10^4^ ha^-1^)	Spikelets per Panicle(grain)	Seed Setting Rate(%)	1000-grain Weight(g)	Yield(kg ha^-1^)
2020	J20	N_0_	195.56 ± 15.39c	137.40 ± 0.54d	74.10 ± 0.06a	22.65 ± 0.07a	4852.17 ± 382.01c
		N_1_	222.22 ± 27.75bc	145.14 ± 0.42c	71.15 ± 0.08b	22.78 ± 0.09a	5633.77 ± 703.66bc
		N_2_	266.66 ± 23.09a	161.37 ± 0.35b	69.16 ± 0.03c	21.83 ± 0.03b	7004.91 ± 606.64a
		N_3_	253.33 ± 13.34ab	175.92 ± 0.13a	64.72 ± 0.06d	21.33 ± 0.05c	6630.96 ± 349.00ab
		N_4_	240.00 ± 26.67ab	176.41 ± 0.51a	63.36 ± 0.10e	20.96 ± 0.09d	6053.73 ± 672.64ab
		CV (%)	11.78	11.12	6.51	3.64	14.00
	Koshihikari	N_0_	226.67 ± 13.34b	97.10 ± 1.26e	87.85 ± 0.16a	24.94 ± 0.37a	5192.19 ± 305.43c
		N_1_	262.22 ± 7.70b	100.04 ± 0.50d	85.40 ± 0.50b	24.59 ± 0.03b	5933.07 ± 174.18bc
		N_2_	337.78 ± 33.55a	101.62 ± 0.17c	80.40 ± 0.03c	24.63 ± 0.05b	7321.02 ± 727.27a
		N_3_	333.33 ± 35.28a	105.12 ± 0.07b	77.17 ± 0.02d	22.94 ± 0.02c	6679.78 ± 706.92ab
		N_4_	324.44 ± 33.56a	111.06 ± 0.07a	72.64 ± 0.02e	22.86 ± 0.02c	6443.87 ± 666.44ab
		CV (%)	16.75	5.20	7.61	4.19	12.69
2021	J20	N_0_	191.11 ± 7.70b	138.22 ± 0.23e	77.12 ± 0.04a	23.45 ± 0.05a	5145.29 ± 207.25c
		N_1_	240.00 ± 13.33a	140.73 ± 0.30d	70.78 ± 0.07b	22.87 ± 0.07b	5887.16 ± 327.06bc
		N_2_	275.56 ± 15.39a	153.11 ± 0.28c	68.35 ± 0.06c	22.75 ± 0.06b	7065.51 ± 394.77a
		N_3_	262.22 ± 20.37a	166.49 ± 0.48b	65.33 ± 0.10d	22.27 ± 0.10c	6841.46 ± 531.38a
		N_4_	244.44 ± 33.55a	178.78 ± 0.58a	62.45 ± 0.12e	21.42 ± 0.11d	6295.04 ± 864.12ab
		CV (%)	13.25	11.07	8.15	3.37	12.32
	Koshihikari	N_0_	240.00 ± 13.33c	99.99 ± 0.21d	85.43 ± 0.03a	26.01 ± 0.06b	5743.57 ± 319.09c
		N_1_	266.67 ± 40.00bc	100.30 ± 0.35d	82.93 ± 0.06b	26.19 ± 0.11a	6255.38 ± 938.31bc
		N_2_	337.78 ± 33.55a	103.71 ± 0.12c	76.62 ± 0.03c	25.58 ± 0.04c	7393.49 ± 734.47a
		N_3_	324.44 ± 7.70a	108.75 ± 0.28b	72.52 ± 0.07d	25.36 ± 0.09d	6988.02 ± 165.80ab
		N_4_	315.56 ± 27.76ab	113.29 ± 0.30a	68.30 ± 0.09e	25.52 ± 0.10c	6710.89 ± 590.27abc
		CV (%)	14.02	5.45	9.21	1.36	9.68
F-value	Year (Y)		0.32ns	54.13**	2118.01**	1841.75**	3.19ns
	Varieties (V)		83.21**	238696.06**	86117.84**	8995.97**	5.06*
	N level (N)		27.61**	8623.86**	21760.71**	497.31**	22.21**
	Y×V		0.32ns	768.36**	2998.84**	391.52**	0.10ns
	Y×N		0.13ns	122.41**	218.88**	40.94**	0.15ns
	V×N		1.96ns	2432.46**	691.00**	19.35**	0.19ns
	Y×V×N		0.25ns	134.20**	86.82**	54.55**	0.02ns

CV represents coefficient of variation. N_0_, N_1_, N_2_, N_3_ and N_4_ refer to the different fertilizer treatments (0, 90, 135, 180 and 225 kg ha^-1^, respectively). Different lowercase letters of the same variety in the same column showed significant differences at the level of P < 0.05, ns indicates no significant difference, * and ** indicate significant difference at 0.05 and 0.01 levels, respectively.

The effective panicle number of the two varieties first increased and then decreased with increasing N and the maximum values were observed under the N_2_ treatment, and the differences between the N_2_, N_3_, and N_4_ treatments and the N_0_ treatment were significant. The number of spikelets per panicle increased with increasing N, and the difference between the N_4_ and N_0_ treatments was significant. The seed setting rate and 1000-grain weight showed the same trend of both decreasing with increasing N, and the difference between the N_0_ treatment and the other treatments was significant.

### 3.2 Processing quality and appearance quality

The processing quality of both rice varieties had the same response to the N application rate tending to deteriorate with increasing N (**Table 3**). The brown rice rate, milled rice rate and head rice rate first increased and then decreased with increasing N and the maximum values were observed under the N_2_ treatment. In the two-year experiment, the coefficient of variation of the head rice rate was the largest among all indicators. The coefficient of variation of the head rice rate of J20 in 2021 was significantly larger than that of Koshihikari, and this indicator was the most sensitive to N fertilization. In terms of appearance quality, the chalky grain rate and chalkiness degree of both varieties first decreased and then increased with increasing N. The minimum values were observed under the N_2_ treatment, which were significantly different from the N_0_ treatment. In the two-year experiment, the coefficient of variation of the chalky grain rate and chalkiness was the largest for Koshihikari, and these parameters were the most sensitive to N fertilization.

**Table 3:** Effects of N application rate on processing quality and appearance quality of rice.

Year	Variety	Treatment	Brown Rice Rate(%)	Milled Rice Rate(%)	Head Rice Rate(%)	Chalky Grain Rate(%)	Chalkiness(%)
2020	J20	N_0_	75.07 ± 0.58c	65.57 ± 0.30a	54.33 ± 0.31c	86.90 ± 1.95a	50.43 ± 2.04a
		N_1_	77.27 ± 0.31ab	66.73 ± 0.12a	55.07 ± 0.31bc	85.43 ± 1.57ab	48.80 ± 1.11ab
		N_2_	77.67 ± 0.12a	67.01 ± 0.06a	56.33 ± 1.10ab	84.30 ± 1.21b	47.43 ± 1.07b
		N_3_	77.47 ± 0.23a	66.85 ± 1.92a	56.27 ± 0.64ab	84.80 ± 0.62ab	47.83 ± 0.75b
		N_4_	76.73 ± 0.42b	66.70 ± 0.24a	56.60 ± 1.04a	85.13 ± 0.85ab	48.03 ± 1.56ab
		CV (%)	1.37	0.86	1.75	1.15	2.45
	Koshihikari	N_0_	78.67 ± 0.58b	70.06 ± 1.14b	52.00 ± 1.11b	15.27 ± 2.24c	5.23 ± 0.84c
		N_1_	80.53 ± 0.70a	72.81 ± 1.35a	53.00 ± 0.72ab	30.17 ± 4.40a	14.07 ± 1.78a
		N_2_	81.47 ± 0.95a	73.34 ± 0.57a	53.40 ± 0.53a	15.83 ± 2.40c	5.83 ± 0.90c
		N_3_	80.47 ± 1.01a	73.13 ± 0.25a	53.07 ± 0.50ab	22.63 ± 1.50b	11.70 ± 1.28b
		N_4_	80.20 ± 0.69a	72.01 ± 0.77a	52.33 ± 0.61ab	21.43 ± 0.67b	10.23 ± 0.60b
		CV (%)	1.26	1.85	1.09	28.73	40.43
2021	J20	N_0_	75.44 ± 0.19c	64.99 ± 0.32c	54.46 ± 1.14d	69.60 ± 3.22a	39.43 ± 2.12a
		N_1_	77.91 ± 0.36b	67.43 ± 0.96b	56.99 ± 0.16c	67.43 ± 2.86ab	36.43 ± 2.03ab
		N_2_	79.30 ± 0.25a	69.27 ± 0.71a	63.83 ± 0.44a	63.73 ± 1.50b	32.27 ± 0.90b
		N_3_	78.02 ± 0.41b	67.15 ± 0.91b	60.13 ± 0.88b	66.43 ± 2.42ab	34.73 ± 3.81ab
		N_4_	77.53 ± 0.34b	67.01 ± 0.49b	57.79 ± 0.99c	67.03 ± 0.15ab	35.27 ± 4.71ab
		CV (%)	1.80	2.26	6.03	3.16	7.33
	Koshihikari	N_0_	75.85 ± 0.41c	66.21 ± 0.45c	54.96 ± 0.92c	27.13 ± 3.80a	9.70 ± 1.87a
		N_1_	78.68 ± 0.18b	68.41 ± 0.56b	57.49 ± 0.45b	18.47 ± 0.46c	7.60 ± 0.70b
		N_2_	80.57 ± 0.33a	69.95 ± 0.97a	60.41 ± 0.96a	18.10 ± 0.56c	7.30 ± 0.20b
		N_3_	80.04 ± 0.47a	68.29 ± 1.26b	58.59 ± 0.54b	26.63 ± 0.78ab	7.53 ± 0.57b
		N_4_	78.61 ± 0.22b	68.73 ± 0.33ab	57.62 ± 0.31b	22.60 ± 3.10b	9.73 ± 0.32a
		CV (%)	2.33	1.98	3.42	19.05	14.70
F-value	Year (Y)		7.70**	67.18**	508.20**	230.76**	258.16**
	Varieties (V)		288.60**	279.38**	106.73**	9460.41**	5864.77**
	N level (N)		74.12**	25.60**	71.24**	10.27**	7.83**
	Y×V		76.04**	123.37**	32.36**	320.98**	186.77**
	Y×N		4.51**	2.31ns	28.11**	12.20**	6.02**
	V×N		0.63ns	0.52ns	6.35**	4.98**	6.64**
	Y×V×N		1.99ns	1.31ns	3.90**	11.49**	5.33**

CV represents coefficient of variation. N_0_, N_1_, N_2_, N_3_ and N_4_ refer to the different fertilizer treatments (0, 90, 135, 180 and 225 kg ha^-1^, respectively). Different lowercase letters of the same variety in the same column showed significant differences at the level of P < 0.05, ns indicates no significant difference, ** indicates significant difference at 0.01 levels.

### 3.3 RVA profile characteristics of brown rice flour starch

The effect of N application on the RVA profile characteristics of rice is shown in **Table 4**. The peak viscosity, trough viscosity, breakdown viscosity and final viscosity of J20 decreased with increasing N (except trough viscosity in 2021), while Koshihikari showed the trend of first increasing and then decreasing. These J20 indexes under the N_2_ treatment were significantly different from those under the N_3_ and N_4_ treatments. As more N was gradually applied, the setback viscosity of J20 first increased and then decreased, while Koshihikari showed the opposite trend. The pasting temperature of J20 showed an overall increasing trend, and Koshihikari first decreased and then increased. All indexes of Koshihikari performed best under the N_2_ treatment, and the difference between the N_2_ and N_0_ treatments was significant. The minimum values of setback viscosity and pasting temperature of J20 were observed under the N_0_ treatment. For both varieties, the coefficient of variation of the setback value was the largest, whereas the coefficient of variation of the peak viscosity and breakdown value of J20 was significantly larger than that of Koshihikari, and these parameters were more sensitive to N fertilization.

**Table 4:** Effects of N application rate on RVA profile characteristics of brown rice flour starch.

Year	Variety	Treatment	Peak Viscosity(RVU)	Trough Viscosity(RVU)	Breakdown Viscosity(RVU)	Final Viscosity(RVU)	Setback Viscosity(RVU)	Pasting Temperature(°C)
2020	J20	N_0_	193.38 ± 1.00a	111.84 ± 5.18a	81.54 ± 4.19a	207.13 ± 4.31a	13.76 ± 3.30b	77.63 ± 0.04c
		N_1_	154.25 ± 0.71cd	94.38 ± 4.89b	59.88 ± 4.18bc	181.67 ± 4.01c	27.42 ± 3.30ab	78.08 ± 0.46bc
		N_2_	165.67 ± 8.01b	106.46 ± 4.89ab	59.21 ± 3.13bc	202.96 ± 1.71ab	37.30 ± 9.72a	78.88 ± 0.67b
		N_3_	158.46 ± 3.13bc	97.17 ± 7.19b	61.30 ± 4.07b	192.84 ± 6.60bc	34.38 ± 3.47a	77.38 ± 0.53c
		N_4_	147.88 ± 0.88d	97.25 ± 3.78b	50.63 ± 4.66c	181.75 ± 5.06c	33.88 ± 4.18a	82.03 ± 0.25a
		CV (%)	10.79	7.30	18.28	6.09	32.14	2.40
	Koshihikari	N_0_	229.21 ± 0.41d	146.92 ± 3.54b	82.29 ± 3.13b	227.75 ± 1.77c	-1.46 ± 1.36a	80.18 ± 0.74a
		N_1_	241.71 ± 1.59b	146.80 ± 2.30b	94.91 ± 0.71a	235.28 ± 3.96ab	-6.43 ± 2.37ab	77.55 ± 1.06bc
		N_2_	251.92 ± 2.35a	154.54 ± 1.82a	97.38 ± 4.18a	239.84 ± 0.59a	-12.08 ± 2.94b	77.28 ± 0.46c
		N_3_	236.92 ± 2.12c	146.46 ± 0.76b	90.46 ± 2.89ab	230.25 ± 0.35bc	-6.67 ± 2.47ab	79.65 ± 0.35ab
		N_4_	235.46 ± 1.24c	150.00 ± 4.36ab	85.46 ± 5.59b	231.79 ± 1.36bc	-3.67 ± 2.60a	78.53 ± 1.24abc
		CV (%)	3.54	2.31	6.99	2.02	65.79	1.61
2021	J20	N_0_	186.09 ± 0.47a	128.00 ± 1.06a	58.09 ± 1.53a	245.00 ± 0.59ab	58.92 ± 1.07b	79.35 ± 0.07b
		N_1_	157.55 ± 2.30c	104.46 ± 6.89b	53.08 ± 9.19ab	217.08 ± 3.54c	59.55 ± 5.83b	79.38 ± 0.04b
		N_2_	186.13 ± 0.53a	133.92 ± 6.24a	52.21 ± 5.71ab	252.17 ± 7.54a	66.04 ± 7.01ab	82.58 ± 0.53a
		N_3_	165.92 ± 2.71b	125.71 ± 0.41a	40.21 ± 3.13bc	237.88 ± 0.29b	71.96 ± 3.01a	80.73 ± 1.03b
		N_4_	158.25 ± 1.77c	123.54 ± 3.48a	34.71 ± 5.25c	226.84 ± 3.77c	68.59 ± 2.00ab	79.65 ± 0.35b
		CV (%)	8.41	9.04	20.51	5.95	8.74	1.71
	Koshihikari	N_0_	240.80 ± 1.59c	152.34 ± 2.35a	88.46 ± 3.95b	264.75 ± 1.30b	23.96 ± 2.89b	81.88 ± 0.46a
		N_1_	248.75 ± 2.47b	157.67 ± 1.89a	91.09 ± 0.59ab	269.96 ± 0.76ab	21.21 ± 1.71b	79.60 ± 0.35b
		N_2_	258.67 ± 1.65a	160.50 ± 6.25a	98.17 ± 4.60a	273.59 ± 3.77a	14.92 ± 2.12c	78.38 ± 0.04c
		N_3_	219.83 ± 3.54e	132.25 ± 4.84b	87.59 ± 1.29b	252.42 ± 3.42c	32.59 ± 0.12a	81.05 ± 0.50a
		N_4_	231.13 ± 1.00d	141.83 ± 3.18b	89.30 ± 2.19b	256.75 ± 2.72c	25.63 ± 1.72b	78.93 ± 0.74bc
		CV (%)	6.29	7.88	4.68	3.36	27.23	1.83
F-value	Year (Y)		22.73**	71.63**	26.80**	1072.20**	666.67**	56.56**
	Varieties (V)		8069.79**	819.24**	683.51**	913.53**	918.65**	1.93ns
	N level (N)		126.59**	17.24**	12.12**	36.52**	7.69**	5.14**
	Y×V		14.29**	71.99**	33.44**	29.01**	5.51*	0.30ns
	Y×N		13.64**	1.56ns	1.27ns	1.54ns	2.22ns	10.74**
	V×N		90.92**	11.81**	14.54**	27.52**	10.28**	28.39**
	Y×V×N		23.16**	9.39**	3.00*	4.49*	2.09ns	6.39**

CV represents coefficient of variation. N_0_, N_1_, N_2_, N_3_ and N_4_ refer to the different fertilizer treatments (0, 90, 135, 180 and 225 kg ha^-1^, respectively). Different lowercase letters of the same variety in the same column showed significant differences at the level of P < 0.05, ns indicates no significant difference, * and ** indicate significant difference at 0.05 and 0.01 levels, respectively.

### 3.4 Protein, protein component and GABA contents in brown rice flour

**Figure 4:**
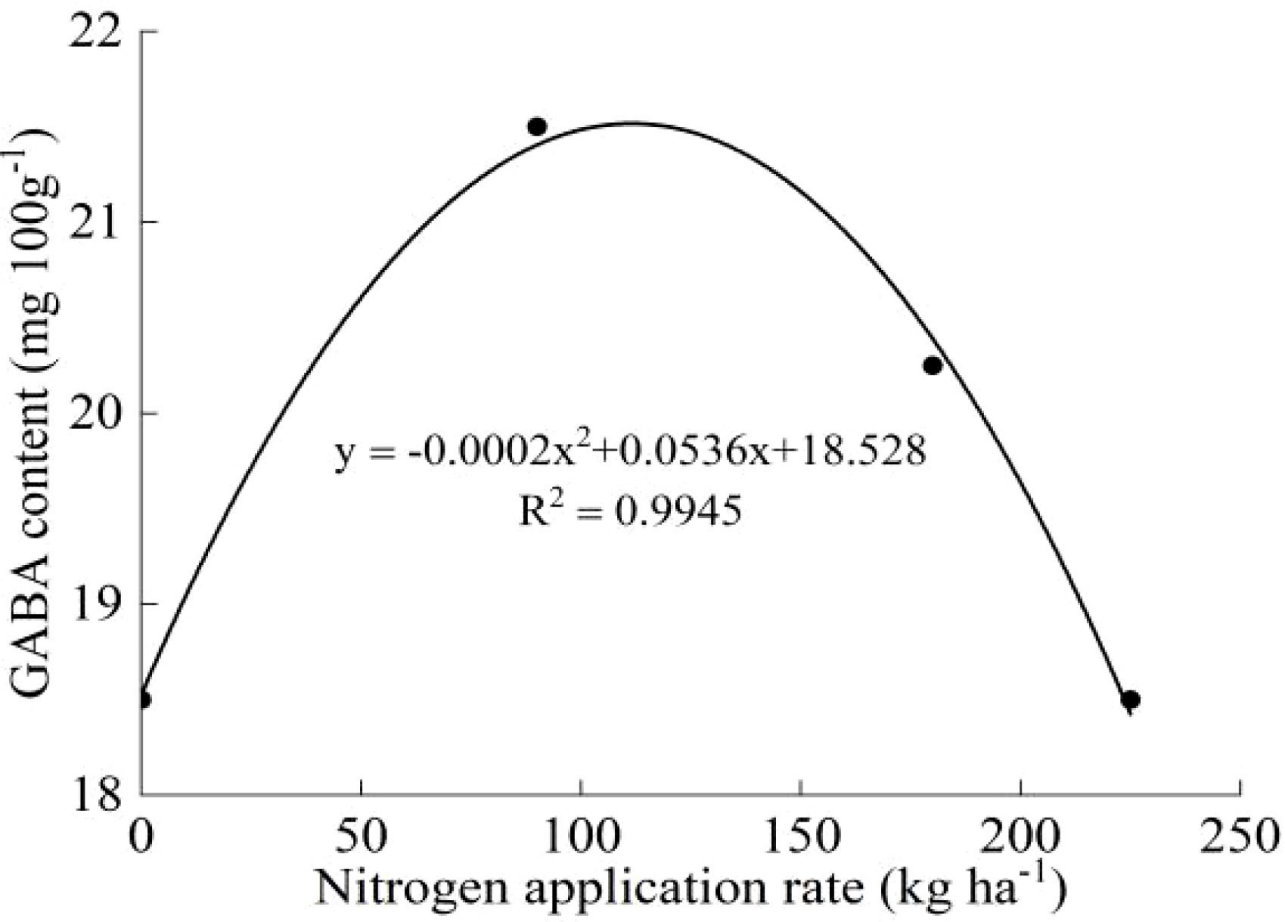
Relationship between J20 GABA content and nitrogen application rate.

**Figure 5:**
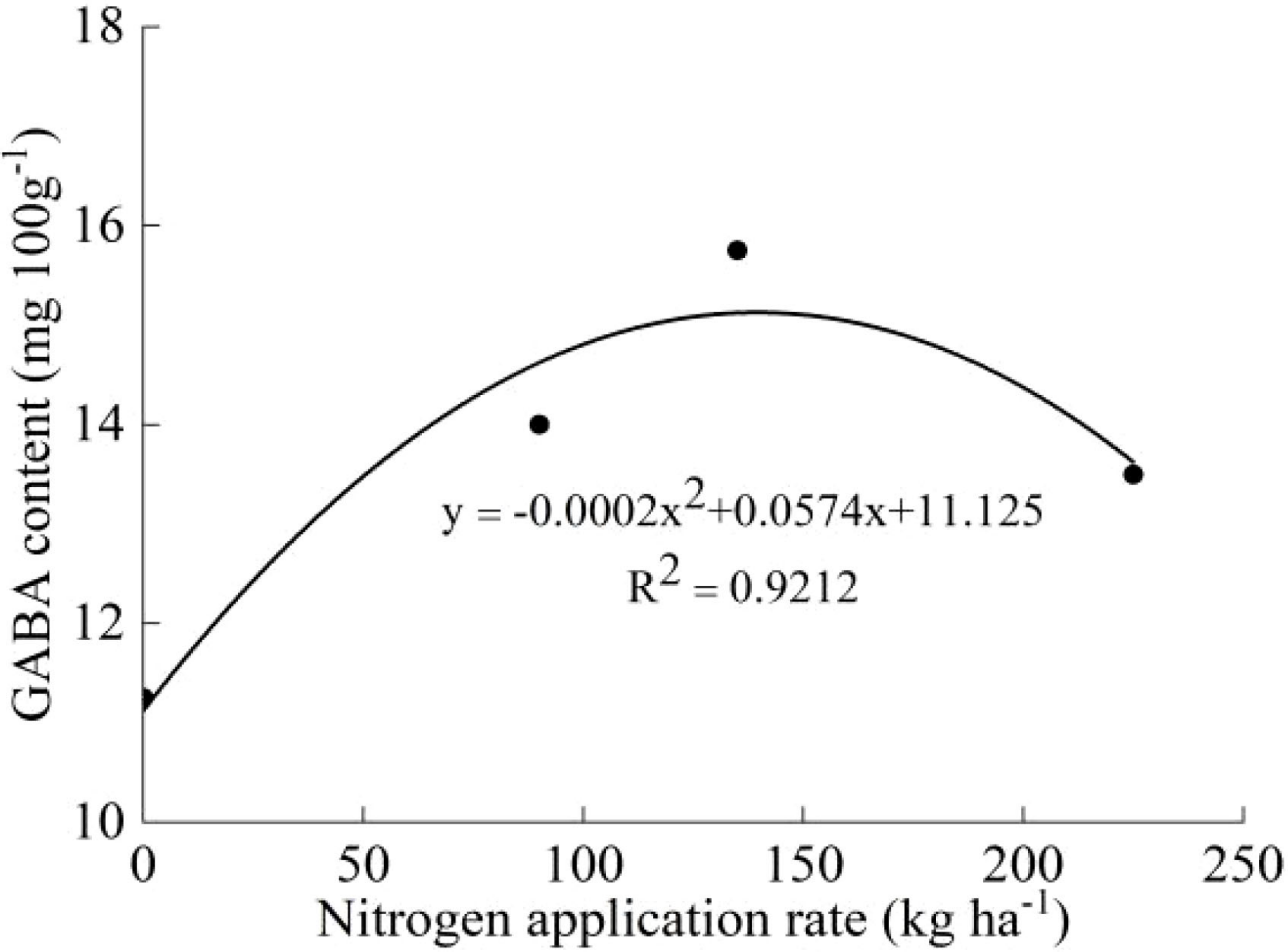
Relationship between Koshihikari GABA content and nitrogen application rate.

The protein contents of both varieties increased with increasing N, and the protein contents under the N_4_ treatment were significantly different from those under the N_0_ treatment (except Koshihikari in 2020) (**Table 5**). The GABA contents of both varieties first increased and then decreased with increasing N, and the maximum values were observed under the N_2_ treatment. No significant differences were found among the treatments (except J20 in 2021). The protein and GABA contents of J20 were higher than those of Koshihikari under the same N application rate. The average GABA content of J20 in the two years was significantly higher than that of Koshihikari which increased by 51.91%. According to the GABA content and nitrogen application rate of J20 and Koshihikari, the effect equations were established. The optimum nitrogen application rate for J20 was 134 kg ha^-1^, and the maximum GABA content was 22.12 mg 100 g^-1^ (**Figure 4**). The optimum nitrogen application rate for Koshihikari was 143.50 kg ha^-1^, and the maximum GABA content was 15.24 mg 100 g^-1^ (**Figure 5**).

**Table 5:** Effects of N application rate on protein content and GABA content in brown rice flour.

Year	Treatment	Protein content (%)		GABA content (mg 100 g^-1^)	
		J20	Koshihikari	J20	Koshihikari
2020	N_0_	8.36 ± 0.13b	8.24 ± 0.04a	18.50 ± 2.12a	11.50 ± 2.12a
	N_1_	8.42 ± 0.04b	8.39 ± 0.08a	21.50 ± 0.71a	14.00 ± 2.83a
	N_2_	8.84 ± 0.29ab	8.42 ± 0.04a	23.50 ± 2.12a	16.50 ± 0.71a
	N_3_	8.90 ± 0.46ab	8.54 ± 0.04a	21.00 ± 1.41a	13.00 ± 2.83a
	N_4_	9.22 ± 0.08a	8.96 ± 0.63a	19.00 ± 2.83a	14.50 ± 0.71a
	CV (%)	4.09	3.21	9.75	13.31
2021	N_0_	8.42 ± 0.04d	8.27 ± 0.08d	18.50 ± 0.71bc	11.00 ± 1.41a
	N_1_	8.60 ± 0.13cd	8.42 ± 0.13cd	21.50 ± 0.71b	14.00 ± 2.83a
	N_2_	8.87 ± 0.16bc	8.57 ± 0.17bc	25.50 ± 0.71a	15.00 ± 2.83a
	N_3_	8.99 ± 0.08ab	8.75 ± 0.08ab	19.50 ± 2.12bc	14.00 ± 4.24a
	N_4_	9.25 ± 0.21a	8.99 ± 0.08a	18.00 ± 1.41c	12.50 ± 0.71a
	CV (%)	3.69	3.27	14.80	11.77
F-value	Year (Y)	1.47ns		0.27ns	
	Varieties (V)	11.10**		108.77**	
	N level (N)	15.31**		6.91**	
	Y×V	0.01ns		0.14ns	
	Y×N	0.10ns		0.20ns	
	V×N	0.48ns		0.81ns	
	Y×V×N	0.13ns		0.51ns	

CV represents coefficient of variation. N_0_, N_1_, N_2_, N_3_ and N_4_ refer to the different fertilizer treatments (0, 90, 135, 180 and 225 kg ha^-1^, respectively). Different lowercase letters of the same variety in the same column showed significant differences at the level of P < 0.05, ns indicates no significant difference, ** indicates significant difference at 0.01 levels.

The content of protein components of both varieties increased with increasing N, and the albumin contents under the N_4_ treatment were significantly different from those under the N_0_ treatment (**Table 6**). In the two-year experiment, the coefficient of variation of gliadin content was the largest, and the coefficient of variation of albumin content of J20 was larger than that of Koshihikari, which was more sensitive to N fertilizer.

**Table 6:** Effect of N application rate on protein component of brown rice flour.

Year	Variety	Treatment	Albumin (%)	Globulin (%)	Gliadin (%)	Glutenin (%)
2020	J20	N_0_	0.72 ± 0.03c	0.68 ± 0.08a	0.15 ± 0.01b	5.51 ± 0.19a
		N_1_	0.72 ± 0.01c	0.66 ± 0.09a	0.17 ± 0.01ab	5.52 ± 0.44a
		N_2_	0.82 ± 0.01b	0.68 ± 0.06a	0.15 ± 0.03b	5.37 ± 0.50a
		N_3_	0.93 ± 0.01a	0.74 ± 0.11a	0.20 ± 0.01a	5.62 ± 0.30a
		N_4_	0.90 ± 0.01a	0.75 ± 0.12a	0.18 ± 0.01ab	6.20 ± 0.29a
		CV (%)	11.99	5.73	12.48	5.73
	Koshihikari	N_0_	0.81 ± 0.01c	0.53 ± 0.06a	0.14 ± 0.02b	5.18 ± 0.24b
		N_1_	0.82 ± 0.01bc	0.57 ± 0.10a	0.16 ± 0.01b	4.95 ± 0.24b
		N_2_	0.85 ± 0.01ab	0.63 ± 0.08a	0.15 ± 0.03b	6.03 ± 0.16a
		N_3_	0.87 ± 0.02a	0.64 ± 0.10a	0.14 ± 0.02b	6.00 ± 0.15a
		N_4_	0.88 ± 0.02a	0.69 ± 0.13a	0.21 ± 0.01a	6.08 ± 0.17a
		CV (%)	3.60	10.23	18.22	9.55
2021	J20	N_0_	0.68 ± 0.02c	0.78 ± 0.04b	0.14 ± 0.01c	5.60 ± 0.20b
		N_1_	0.69 ± 0.01c	0.79 ± 0.03b	0.16 ± 0.01b	5.61 ± 0.09b
		N_2_	0.81 ± 0.04b	0.79 ± 0.03b	0.14 ± 0.01c	5.87 ± 0.03ab
		N_3_	0.85 ± 0.02b	0.80 ± 0.05b	0.15 ± 0.01bc	5.97 ± 0.30ab
		N_4_	0.92 ± 0.03a	0.96 ± 0.10a	0.19 ± 0.01a	6.20 ± 0.13a
		CV (%)	13.12	9.27	13.29	4.34
	Koshihikari	N_0_	0.80 ± 0.01b	0.63 ± 0.04bc	0.11 ± 0.01c	5.45 ± 0.63a
		N_1_	0.82 ± 0.02b	0.55 ± 0.06c	0.12 ± 0.01bc	5.30 ± 0.20a
		N_2_	0.81 ± 0.01b	0.75 ± 0.02a	0.13 ± 0.01bc	5.37 ± 0.55a
		N_3_	0.81 ± 0.02b	0.76 ± 0.04a	0.16 ± 0.03b	6.14 ± 0.30a
		N_4_	0.86 ± 0.01a	0.73 ± 0.07ab	0.22 ± 0.01a	6.11 ± 0.14a
		CV (%)	2.86	13.30	29.99	7.32
F-value	Year (Y)		21.88**	200.89**	7.50*	2.14ns
	Varieties (V)		24.93**	266.00**	3.50ns	1.19ns
	N level (N)		100.49**	5458**	26.61**	14.44**
	Y×V		0.01ns	10.58**	0.01ns	1.31ns
	Y×N		4.43*	3.06*	1.89ns	0.63ns
	V×N		43.85**	11.98**	4.49*	2.45ns
	Y×V×N		1.93ns	10.04**	3.11*	2.64ns

CV represents coefficient of variation. N_0_, N_1_, N_2_, N_3_ and N_4_ refer to the different fertilizer treatments (0, 90, 135, 180 and 225 kg ha^-1^, respectively). Different lowercase letters of the same variety in the same column showed significant differences at the level of P < 0.05, ns indicates no significant difference, * and ** indicate significant difference at 0.05 and 0.01 levels, respectively.

### 3.5 Correlation analysis

The correlation analysis of J20 indicators is shown in **Table 7** (below the diagonal). The nitrogen application rate had a significant or extremely significant positive correlation with setback value and protein content, while there was a significant or extremely significant negative correlation with 1000-grain weight and breakdown value. The yield was significantly or extremely significantly positively correlated with the head rice rate and setback value but negatively correlated with the chalky grain rate and chalkiness degree. The correlation analysis of Koshihikari indicators is shown in **Table 7** (above the diagonal). The nitrogen application rate was significantly or extremely significantly positively correlated with protein content, while it was significantly negatively correlated with 1000-grain weight. The GABA content was significantly positively correlated with the head rice rate and breakdown value.

**Table 7:** Correlation analysis between yield and quality of J20 (below the diagonal) and Koshihikari (above the diagonal).

Index	N level	1000-grain Weight	Yield	Head Rice Rate	Chalky Grain Rate	Chalkiness	Peak Viscosity	Breakdown Viscosity	Setback Viscosity	Protein Content	GABA Content
N level		-0.889*	0.714	0.558	0.108	0.379	-0.191	0.136	0.073	0.939*	0.514
1000-grain Weight	-0.958*		-0.497	-0.282	-0.300	-0.360	0.582	0.243	-0.460	-0.880*	-0.137
Yield	0.720	-0.580		0.949*	-0.403	-0.190	0.375	0.688	-0.508	0.483	0.878
Head rice rate	0.640	-0.511	0.982**		-0.347	-0.135	0.543	0.854	-0.640	0.269	0.955*
Chalky Grain Rate	-0.628	0.461	-0.961**	-0.982**		0.868	-0.691	-0.503	0.756	0.129	-0.417
Chalkiness	-0.742	0.590	-0.978**	-0.978**	0.987**		-0.439	-0.260	0.494	0.405	-0.091
Peak Viscosity	-0.760	0.632	-0.347	-0.232	0.321	0.423		0.884*	-0.986**	-0.344	0.701
Breakdown Viscosity	-0.981**	0.927*	-0.619	-0.536	0.554	0.671	0.856		-0.912*	-0.133	0.919*
Setback Viscosity	0.917*	-0.823	0.934*	0.875	-0.848	-0.920*	-0.574	-0.843		0.240	-0.773
Protein Content	0.961**	-0.989**	0.654	0.603	-0.559	-0.674	-0.595	-0.922*	0.858		0.264
GABA Content	0.047	0.170	0.653	0.731	-0.794	-0.691	0.051	0.003	0.369	-0.051	

* and ** indicate significant difference at 0.05 and 0.01 levels, respectively.

### 3.6 Body weight analysis of different groups of rats during the feeding cycle

The results showed that there was an increasing trend of body weight with the development of the feeding cycle, but the body weight gain was different among the groups (**Table 8**). No significant difference was found in the starting body weight of rats in the different groups. After 1 week of feeding, compared with the control group, the body weight of the rats in the J20 brown rice group and the Koshihikari brown rice group was significantly reduced, but the difference between the two experimental groups was not significant. After 2 weeks of feeding, the body weight of the rats followed the trend of control group>Koshihikari brown rice group>J20 brown rice group, and there were significant differences among the groups. After 5 weeks of feeding, the body weight of the rats in the two experimental groups decreased by 6.33% (Koshihikari brown rice group) and 10.39% (J20 brown rice group) compared with the control group. These results indicated that both J20 brown rice and Koshihikari brown rice could significantly slow the weight gain of rats, and the effect of J20 brown rice was more obvious.

**Table 8:** Weight analysis of different groups of rats in feeding cycle.

Weeks	Group (g)		
	Control	Koshihikari brown rice	J20 brown rice
0	314.80 ± 1.75a	315.35 ± 1.60a	315.71 ± 1.38a
1	325.52 ± 1.80a	316.26 ± 1.97b	316.79 ± 2.75b
2	340.13 ± 2.40a	323.38 ± 1.07b	318.73 ± 1.97c
3	350.67 ± 1.96a	329.34 ± 1.07b	321.24 ± 1.76c
4	357.68 ± 1.29a	336.34 ± 1.26b	324.13 ± 1.15c
5	365.04 ± 1.45a	341.94 ± 2.02b	327.11 ± 1.34c

Different lowercase letters in different groups of peers are significantly different at the level of P < 0.05.

### 3.7 Detection of serum lipid-related indexes in different groups of rats

After the end of the test period, the detection results of serum lipid-related indexes of rats in each group are shown in **Table 9**. The results showed that the J20 brown rice could significantly reduce the concentrations of serum TG and TC of rats. The HDLC test results showed that the serum HDLC concentration of rats followed the trend of J20 brown rice group > Koshihikari brown rice group > control group. The serum LDLC concentration of rats fed J20 brown rice was the lowest, but no significant difference was found among the groups.

**Table 9:** Detection results of serum lipid related indexes in different groups of rats.

Index	Group (mmol L^-1^)		
	Control	Koshihikari brown rice	J20 brown rice
TG	1.87 ± 0.01a	1.59 ± 0.02b	1.53 ± 0.01c
TC	3.92 ± 0.12a	3.76 ± 0.02a	3.43 ± 0.08b
HDLC	1.84 ± 0.07b	2.05 ± 0.10a	2.12 ± 0.02a
LDLC	1.24 ± 0.05a	1.24 ± 0.01a	1.20 ± 0.02a

TG, TC, HDLC and LDLC represent triglyceride, total cholesterol, high-density lipoprotein cholesterol and low-density lipoprotein cholesterol, respectively. Different lowercase letters in different groups of peers are significantly different at the level of P < 0.05.

## 4 Discussion

### 4.1 Effects of nitrogen application rate on yield of giant embryo rice

N is one of the most important factors limiting rice growth and yield formation. Studies have shown that due to the differences in varieties and regions, the requirements of N application rates for high yields of different rice varieties differ. However, the N application rate does have a parabolic relationship with yield within a certain range (Xu et al., 2011; Cheng et al., 2018). The results of this study showed that as more N was gradually applied, the yield of both varieties first increased and then decreased. The highest yield was observed under the N_2_ treatment. The average yields in the two years of J20 and Koshihikari were 7035.21 kg ha^-1^ and 7357.26 kg ha^-1^, respectively. It is worth noting that in this study, under the same N application rate, the 1000-grain weight and yield of Koshihikari were higher than those of J20, which may be due to the increase in the proportion of embryos, insufficient space for grains to accumulate starch and poor endosperm development of J20 (Zhang, 2008). In addition, the energy consumed by embryo enlargement comes from the starch in rice, and a large reduction in starch leads to a decrease in 1000-grain weight and yield (Du, 2014). The effect equation was established according to the yield and N application rate of the two varieties, and it was calculated that the highest N application rates were 136.22 kg ha^-1^ (J20) and 136.68 kg ha^-1^ (Koshihikari), respectively, which were in good agreement with the actual values. These results indicated that the giant embryo rice J20 could obtain high yield under the conventional fertilizer application rate, and there was no significant difference between Koshihikari. Therefore, farmers might gain higher economic benefits by planting functional rice J20 under conventional field management measures without additional costs.

### 4.2 Effects of nitrogen application rate on the quality of giant embryo rice

In previous studies, the processing quality was found to be better under low N treatment, while the chalky grain rate and chalkiness first decreased and then increased with increasing N, indicating that the appropriate N application rate was conducive to the improvement of appearance quality (Yin et al., 2013). The present study showed that over a certain range, the brown rice rate, milled rice rate and head rice rate of both varieties increased with increasing N, while the chalky grain rate and chalkiness degree showed the opposite trend. However, when the amount of N fertilizer exceeded 135 kg ha^-1^, the processing quality and appearance quality worsened. The chalky grain rate and chalkiness degree of J20 were higher than those of Koshihikari under the same N application rate. We speculated that this was because the abnormal embryo development leads to excessive consumption of the carbon source entering the grain, which affects the starch synthesis in the endosperm. Alternatively, the chalky part was far away from the embryo and the decrease in carbon source transport efficiency led to insufficient filling of starch (Ge et al., 2021).

The characteristics of the starch RVA are closely related to the taste of rice. Rice with better eating quality generally has high peak viscosity, high breakdown viscosity and low setback viscosity (Yang et al., 2020). It is generally believed that the N application rate is negatively correlated with the RVA characteristics of rice starch (Hu et al., 2018; Wen et al., 2020). However, some studies have also found that either too high or too low of an N application rate would make the RVA profile characteristics worse (Guo et al., 2021). This study found that the sensitivity of different rice varieties to N fertilization differed. The cooking and eating quality of J20 was affected by N application more easily than that of Koshihikari. The highest peak viscosity, highest breakdown viscosity and lowest setback viscosity of J20 and Koshihikari were observed under the N_0_ and N_2_ treatments, respectively. The peak viscosity, trough viscosity, breakdown viscosity and final viscosity of J20 under the same N application rate were lower than those of Koshihikari. This result might be due to the higher protein content in J20, which inhibited the combination of water and rice flour, thereby reducing the viscosity (Zhu et al., 2016).

Previous studies have found that increasing the amount of N application can increase the protein content of rice and reduce the amylose content, which may be due to the accumulation of N in the plant and the enhanced activity of protein synthesis-related enzymes, thereby promoting protein synthesis (Zhang et al., 2021). The results of this study found that the contents of protein and protein components of the two varieties increased with increasing N in agreement with the results of previous studies. The coefficient of variation of gliadin was the largest and more sensitive to N fertilizer, and the protein content of J20 was higher than that of Koshihikari under the same nitrogen application, indicating that its nutritional value was higher.

### 4.3 Response of GABA content in giant embryo brown rice to nitrogen application and analysis of lipid-lowering effects

GABA is a naturally occurring non-protein amino acid with a variety of physiological functions, especially in hyperlipidemia and cerebrovascular diseases. Studies have shown that under high-fat diet conditions, feeding giant embryo brown rice can significantly inhibit body weight gain and reduce blood glucose concentration, plasma total cholesterol and triglyceride concentrations in mice (Kang et al., 2013). However, there have been limited studies on the effects of the N application rate on giant embryo rice, especially the GABA content. This study found that the GABA content of both varieties first increased and then decreased with increasing N and the maximum values were observed under N_2_ treatment. Correlation analysis showed that there was a positive correlation between nitrogen application rate and GABA content, but it was not significant. The GABA content of J20 was significantly higher than that of Koshihikari, indicating that J20 was significantly better than common rice in terms of functional characteristics. The effect equation was established according to the GABA content and N application rate of the two varieties, and it was calculated that the N application rates with the highest functional components were 134 kg ha^-1^ (J20) and 143.50 kg ha^-1^ (Koshihikari), respectively, which were close to the actual values. These results indicated that the yield and functional components of J20 could be improved simultaneously with an appropriate N application rate. The highest yield and GABA content of J20 were both observed under the N_2_ treatment. Therefore, as a functional rice richer in nutrients than common cultivated rice but slightly decreased in yield, the giant embryo rice still deserves broad application and promotion prospects. Animal experiments showed that both J20 brown rice and Koshihikari brown rice could significantly slow the weight gain of rats, and the effect of J20 brown rice was more obvious. When compared with the control group, J20 brown rice could significantly reduce the serum total cholesterol and triglyceride content of the rats and significantly increase the high-density lipoprotein cholesterol content, indicating that J20 brown rice had the effect of relieving hyperlipidemia and could become a better dietary choice. It is worth noting that GABA mainly existed in the rice germ, which was often discarded along with the rice bran layer during processing and milling. Due to its poor expansibility and water absorption, ease of cooking, rough taste and difficult digestion and absorption by the human body, brown rice is generally unpopular. Previous studies found that giant germ rice could reduce the serum cholesterol and triglyceride levels in guinea pigs fed a high-fat diet, and it had the effect of weight loss and fat reduction (Zhang, 2011). Therefore, how to improve the cooking, eating and nutritional quality of giant embryo brown rice is worth in-depth study and has become an important problem focused on by an increasing number of scientists.

## 5 Conclusions

Giant embryo rice is a special kind of functional rice. Compared with ordinary rice, a large amount of GABA is found in giant embryo rice, which has anticancer and antihyperlipidemia functions. In recent years, a series of important advances have been made in the research of giant embryo rice. However, there are still limited reports about the effects of fertilizer application rates on giant embryo rice. J20 brown rice slowed the weight gain of rats and reduced the level of hyperlipidemia. Compared with those of Koshihikari, the yield, head rice rate, peak viscosity and breakdown value of J20 were more sensitive to N fertilizer, and the protein content and GABA content of J20 were higher than those of Koshihikari under the same N application rate. The optimal N application rate for achieving high yield, high quality and good functional characteristics in giant embryo rice J20 was 135 kg ha^-1^. The present study indicates that increasing the application rate of N could be used as a simple and easy method to achieve high yield and high GABA content and provides an important reference for the development and application promotion of giant embryo rice.

## Data availability statement

The original contributions presented in the study are included in the article/supplementary material. Further inquiries can be directed to the corresponding authors.

## Ethics statement

The animal study was reviewed and approved by Southwest University of Science and Technology.

## Author contributions

All authors listed have made a substantial, direct, and intellectual contribution to the work and approved it for publication.

## Funding

This study was supported by the Sichuan Science and Technology Program (2020YFH0146).

## Acknowledgments

We would like to thank our teacher for carefully reading and correcting our manuscript and providing technical assistance and financial support for the study, as well as our scientific research team for their contribution to this paper.

## Conflict of interest

The authors declare that the research was conducted in the absence of any commercial or financial relationships that could be construed as a potential conflict of interest.

## Publisher’s note

All claims expressed in this article are solely those of the authors and do not necessarily represent those of their affiliated organizations, or those of the publisher, the editors and the reviewers. Any product that may be evaluated in this article, or claim that may be made by its manufacturer, is not guaranteed or endorsed by the publisher.

## References

[B1] Cheng C. Zeng Y. J. Wang Q. Tan X. M. Shang Q. Y. Zeng Y. H.. (2018). Effects of nitrogen application rate on yield, quality and nitrogen absorption and utilization of late japonica rice yongyou 1538. J. Soil Water Conserv. 32, 222–228. doi: 10.13870/j.cnki.stbcxb.2018.05.036

[B2] Chung S. I. Kang M. Y. Tundis R. (2021). Oral administration of germinated, pigmented, giant embryo rice (*L.* cv. *Keunnunjami*) extract improves the lipid and glucose metabolisms in high-fat diet-fed mice. Oxid. Med. Cell. Longev., 2021 1–9. doi: 10.1155/2021/8829778 PMC7846407 33552386

[B3] Dai H. Y. Zhang R. P. Hua J. S. Cai G. Z. (2011). Effects of different cultivation methods on yield and quality of japonica giant embryo rice. Chin. Agric. Sci. Bulletin 27, 179–183.

[B4] Du B. (2014). Study on embryo growth dynamics and nutritional components of giant embryo rice (Yanji, Jilin: Master's thesis of Yanbian University).

[B5] Fang H. M. Ge C. C. Zhang J. Zhang L. (2021). Gene cloning and functional research progress of giant embryo mutant in rice. Mol. Plant Breed. 19, 7092–7100. doi: 10.13271/j.mpb.019.007092

[B6] Ge X. Y. Liu S. J. Li X. Yan H. G. Wang Y. X. Fu Y. S.. (2021). Study on chalkiness formation mechanism of rice giant embryo mutant N2-52. J. Nanjing Agric. Univ. 44, 217–224. doi: 10.7685/jnau.202005010

[B7] Guo T. Wang H. F. Xue F. Fang W. W. Jiang Y. F. Zhang H. X.. (2021). Effects of topdressing amount of nitrogen fertilizer on quality and RVA spectrum eigenvalues of starch of fragrant rice 1601. North Rice 51, 6–10. doi: 10.16170/j.cnki.1673-6737.2021.01.002

[B8] Hee-Ky S. Hye-W S. Eun-S J. Byo-S M. Hwan L. C. Jae-J L. (2020). Gochujang prepared using rice and wheat koji partially alleviates high-fat diet-induced obesity in rats. Food Sci. Nutr. 8, 1562–1573. doi: 10.1002/fsn3.1443 32180965 PMC7063360

[B9] Hu Y. J. Qian H. J. Wu P. Zhu M. Xing Z. P. Dai Q. G.. (2018). Effects of the amount of nitrogen, phosphorus and potassium on the yield and quality of soft rice japonica rice under the condition of straw returning. J. Plant Nutr. Fert 24, 817–824. doi: 10.11674/zwyf.17347

[B10] Ju C. X. Buresh R. J. Wang Z. Q. Zhang H. Liu L. J. Yang J. C.. (2015). Root and shoot traits for rice varieties with higher grain yield and higher nitrogen use efficiency at lower nitrogen rates application. Field Crops Res. 175, 47–55. doi: 10.1016/j.fcr.2015.02.007

[B11] Kang M. Y. Moon J. E. Lee S. C. (2013). Modulatory effects of functional rice cultivars giant embryo and aranghyangchal on the body weight and lipid metabolism in mice fed with a high fat diet. J. Crop Sci. Biotechnol. 16, 167–171. doi: 10.1007/s12892-013-0055-0

[B12] Kawakami K. Yamada K. Yamada T. Nabika T. Nomura M. (2018). Antihypertensive effect of γ-aminobutyric acid-enriched brown rice on spontaneously hpertensive rats. J. Nutr. Sci. Vitaminol 64, 56–62. doi: 10.3177/jnsv.64.56 29491273

[B13] Lan Y. Sui X. D. Wang J. Wu C. Y. Ding C. B. Li T. (2021). Optimization of protein extraction process of low gluten rice components. J. Chin. Cereals Oils Assoc. 36, 107–113+127. doi: 10.3969/j.issn.1003-0174.2021.12.017

[B14] Pan J. F. Liu Y. Z. Zhong X. H. Lampayan M. R. Singleton R. G. Huang N. G.. (2017). Grain yield, water productivity and nitrogen use efficiency of rice under different water management and fertilizer-n inputs in south China. Agric. Water Manage. 184, 191–200. doi: 10.1016/j.agwat.2017.01.013

[B15] Peng B. Xu K. He K. Tang D. Y. Peng J. Tian X. Y.. (2019). Genetic basis of giant embryo traits and effects of environmental factors on giant embryo rice. J. Mol. Biol. Res. 9, 149. doi: 10.5539/jmbr.v9n1p149

[B16] Ren Y. G. Zhang J. Z. Zhang H. M. Bai J. Liu D. J. Li J. Y. (2011). New giant embryo rice varieties obtained by *in vitro* culture of mature embryos and their characters and rice quality analysis. J. Shanghai Normal Univ. (Natural Sci. Edition) 40, 289–294. doi: 10.3969/j.issn.1000-5137.2011.03.013

[B17] Wei Z. C. Zhang M. W. Chi J. W. Xu Z. H. Zhang Y. Li J. X.. (2005). Analysis and comparison of rice quality and nutritional components between introduced giant embryo rice and ordinary rice. J. Plant Genet. Resour. 4, 386–389. doi: 10.13430/j.cnki.jpgr.2005.04.005

[B18] Wen C. Y. Xiong Y. H. Yao X. Y. Chen C. L. Hu B. L. Huang Y. P.. (2020). Effects of nitrogen fertilizer application on rice quality and yield of special rice flour. Chin. J. Rice Sci. 34, 574–585. doi: 10.16819/j.1001-7216.2020.0504

[B19] Xu C. M. Zhou C. N. Zheng G. S. Wang D. Y. Hu P. S. Zhang X. F. (2011). Effects of nitrogen application rate and cultivation density on nitrogen accumulation, transport and absorption and utilization efficiency in different organs of super early rice. Soil Fert Sci. China 1, 15–20. doi: 10.3969/j.issn.1673-6257.2011.01.004

[B20] Yang Y. L. (2008). Analysis of nutritional components and germination test of high quality giant embryo rice (Fuzhou, Fujian: Master's thesis of Fujian agriculture and Forestry University).

[B21] Yang T. T. Xie J. X. Huang S. Tan X. M. Pan X. H. Zeng Y. J.. (2020). Effects of post anthesis warming on yield and rice quality of double cropping late japonica rice. Sci. Agric. Sin. 53, 1338–1347. doi: 10.3864/j.issn.0578-1752.2020.07.004

[B22] Yin C. Y. Wang S. Y. Liu H. M. Xue Y. Z. Zhang X. Wang H. L.. (2013). Effects of nitrogen application rate on strong and weak grain filling and rice quality of super japonica rice xindao 18. Chin. J. Rice Sci. 27, 503–510. doi: 10.3969/j.issn.1001-7216.2013.05.007

[B23] Zhang L. L. (2008). Characteristics of giant embryo mutation in rice and metabolic spectrum of grain filling and germination (Hangzhou, Zhejiang: Doctoral Dissertation of Zhejiang University).

[B24] Zhang T. Q. (2011). Preventive study of giant embryo rice on hyperlipidemia in guinea pigs (Shanghai: Master's thesis of Shanghai Normal University).

[B25] Zhang Q. Q. Chen J. Y. Huang R. H. Zhang S. B. (2008). Anatomical observation on embryo development of giant embryo rice. J. Nucl. Agric. Sci. 2, 122–126.

[B26] Zhang Q. Guo B. W. Hu Y. J. Zhang H. C. Xu Y. F. Xu X. J.. (2021). Yield and quality differences of high-quality and high-yield soft rice japonica rice under different nitrogen fertilizer levels. Chin. J. Rice Sci. 35, 1–15. doi: 10.16819/j.1001-7216.2021.201101

[B27] Zhang X. Ping B. Z. Zhang Y. X. Wang M. Li J. Y. (2017). Establishment of molecular markers of giant embryo gene in "Shangshida no. 5" giant embryo rice. Mol. Plant Breed. 15, 4512–4517. doi: 10.13271/j.mpb.015.004512

[B28] Zhang Y. H. Zheng Z. Chen Y. Y. Huang R. H. Zheng B. D. Zhang Q. Q. (2013). Analysis of nutritional components of new giant embryo rice lines. J. Nucl. Agric. Sci. 27, 1331–1336. doi: 10.11869/hnxb.2013.09.1331

[B29] Zheng Z. Huang Z. C. Zhang Y. H. Huang R. H. Zhang Q. Q. (2012). Research progress on giant embryo rice. Subtropical Agric. Res. 8, 221–225. doi: 10.13321/j.cnki.subtrop.agric.res.2012.04.007

[B30] Zhu D. W. Zhang H. C. Guo B. W. Xu K. Dai Q. G. Wei C. X.. (2016). Effect of nitrogen management on the structure and physicochemical properties of rice starch. J. Agric. Food Chem. 64, 8019–8025. doi: 10.1021/acs.jafc.6b03173 27715058

